# Predicting early refractoriness of transarterial chemoembolization in patients with hepatocellular carcinoma using a random forest algorithm: A pilot study

**DOI:** 10.7150/jca.63370

**Published:** 2021-10-17

**Authors:** Zhi-Min Zou, Tian-Zhi An, Jun-Xiang Li, Zi-Shu Zhang, Yu-Dong Xiao, Jun Liu

**Affiliations:** 1 Department of Radiology, the Second Xiangya Hospital of Central South University, Changsha, 410011, China.; 2 Clinical Research Center for Medical Imaging in Hunan Province, Changsha, 410011, China.; 3 Department of Radiology Quality Control Center, Changsha, 410011, China.; 4 Department of Interventional Radiology, the Affiliated Hospital of Guizhou Medical University, Guiyang, 550002, China.; 5 Department of Interventional Radiology, Guizhou Medical University Affiliated Cancer Hospital, Guiyang, 550004, China.; 6 Department of Radiology, Hunan Children's Hospital, Changsha, 410007, China.

**Keywords:** Hepatocellular Carcinoma, Transarterial Chemoembolization, Refractoriness, Predictive Model, Random Forest

## Abstract

**Purpose:** To develop and validate a random forest (RF) based predictive model of early refractoriness to transarterial chemoembolization (TACE) in patients with unresectable hepatocellular carcinoma (HCC).

**Methods:** A total of 227 patients with unresectable HCC who initially treated with TACE from three independent institutions were retrospectively included. Following a random split, 158 patients (70%) were assigned to a training cohort and the remaining 69 patients (30%) were assigned to a validation cohort. The process of variables selection was based on the importance variable scores generated by RF algorithm. A RF predictive model incorporating the selected variables was developed, and five-fold cross-validation was performed. The discrimination and calibration of the RF model were measured by a receiver operating characteristic (ROC) curve and the Hosmer-Lemeshow test.

**Results:** The potential variables selected by RF algorithm for developing predictive model of early TACE refractoriness included patients' age, number of tumors, tumor distribution, platelet count (PLT), and neutrophil-to-lymphocyte ratio (NLR). The results showed that the RF predictive model had good discrimination ability, with an area under curve (AUC) of 0.863 in the training cohort and 0.767 in the validation cohort, respectively. In Hosmer-Lemeshow test, the RF model had a satisfactory calibration with P values of 0.538 and 0.068 in training cohort and validation cohort, respectively.

**Conclusion:** The RF algorithm-based model has a good predictive performance in the prediction of early TACE refractoriness, which may easily be deployed in clinical routine and help to determine the optimal patient of care.

## Introduction

Hepatocellular carcinoma (HCC) is one of the most common malignancies in the alimentary system 1. Transarterial chemoembolization (TACE) is a standard of care for intermediate-stage HCC 1-3. However, not all HCC patients can respond well to TACE because the patients selected for TACE correspond to a highly heterogeneous population, covering a wide range of tumor burden, liver function, and treatment history, and some patients even show TACE failure at the very beginning of their treatment 4, 5. It is recommended that HCC patients with TACE refractoriness should switch to systemic therapy as soon as possible, because repeat TACE is no longer beneficial for such patients 6, 7. Thus, appropriate judgement of TACE refractoriness is crucial. Several previous studies have reported predictive factors of TACE refractoriness 8, 9, however, the potential prediction of early TACE refractoriness have not been identified.

With the development of machine learning (ML) algorithms, an increasing number of predictive models have been established for predicting the therapeutic outcome for HCC patients. ML algorithms can simulate human learning to detect hidden patterns within the data, which is showed a better predictive performance over the traditional statistical method. Random forest (RF) is regarded as one of the most promising ML algorithm 10, and it is consisted of an ensemble learning approach of multiple unique decision trees. Although RF algorithm has previously been utilized to predict the prognosis of HCC patients after various treatment modalities 11-15, it has not yet been used to predict the early TACE refractoriness. Therefore, the purpose of the present study is to develop and validate a predictive model of early TACE refractoriness based on an RF algorithm.

## Materials and methods

### Patients

This retrospective study was approved by the institutional review board of the Second Xiangya Hospital and was performed in accordance with the Declaration of Helsinki. The requirement for written informed consent was waived by the institutional review boards due to the retrospective nature of the present study.

**Figure 1:**
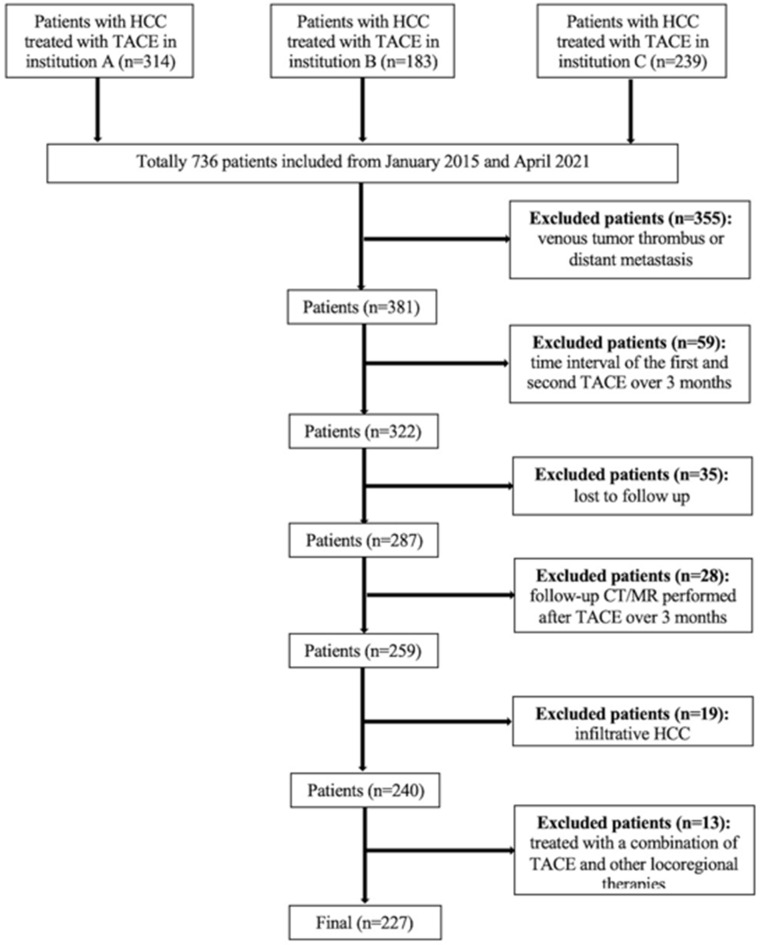
Flowchart of the study population.

A total of 736 consecutive patients with unresectable HCC who underwent TACE at three institutions between January 2015 and April 2021 were included. According to the Barcelona-Clinic-Liver-Cancer (BCLC) staging system, TACE is recommended as an alternative treatment for patients with BCLC-A or a standard treatment for patients with BCLC-B, therefore, the inclusion criteria and exclusion criteria are made as BCLC staging system suggested. The inclusion criteria were as follows: (1) compensated liver function (Child-Pugh class A or B); (2) Eastern Cooperative Oncology Group (ECOG) criteria score of 0; and (3) at least two consecutive TACE sessions performed, or although only one TACE session performed, complete response (CR) achieved after the procedure. The exclusion criteria were as follows: (1) patients for whom had portal venous tumor thrombus (n=236); (2) patients for whom had distant metastasis (n=119); (3) patients for whom the interval between the first and second TACE sessions was longer than 3 months (n=59); (4) patients lost to follow-up (n=35); (5) patients for whom follow-up computed tomography (CT) or magnetic resonance (MR) imaging was performed more than 3 months after TACE (n=28); (6) patients with infiltrative HCC (n=19); and (7) patients who were initially treated with a combination of TACE and other locoregional therapies such as ablation (n=13). The flowchart of the study population is shown in Figure 1.

### TACE procedure

The TACE procedures were discussed with the tumor board prior to administration for each patient. Celiac trunk and superior mesenteric arteriography, as well as indirect portography, were performed to visualize the variations in hepatic arterial anatomy and to evaluate the patency of the portal vein. Either a 2.2 French (Carnelian, Tokai Medical Products, Japan) or a 2.7 French (Progreat, Terumo Medical Corporation, Japan) coaxial microcatheter was placed into the tumor-feeding arteries with the assistance of cone beam computed tomography (CBCT) if needed. Chemoembolization was performed using either up to 15 ml emulsion of iodized oil (Lipiodol, Guerbet, France) mixed with epirubicin (Shandong New Time Pharmaceutical Co., Ltd., China) or drug-eluting beads (DEB) (CalliSpheres Beads, Jiangsu Hengrui Medicine Co., Ltd., China) loaded with epirubicin. The oil-epirubicin emulsion was created using the water-in-oil technique by mixing iodized oil with a distilled water solution containing a drug cocktail of dissolved epirubicin at a ratio of 3:1. The dosage of epirubicin in conventional TACE was 50-75 mg/m2 body surface area, while in DEB-TACE, the dosage of epirubicin ranged from 50 to 150 mg. In conventional TACE, gelfoam slurries were injected to embolize the proximal tumor feeders after the oil-epirubicin emulsion was injected, while in DEB-TACE, no additional embolization was performed. The size of DEBs varied from 100-300 um and 300-500 um. The technical endpoint of TACE was defined as the reduction in arterial inflow to the tumor and tumor devascularization. Changes in chemotherapy drugs, embolic agents, or tumor-feeding artery reselection were made for the second TACE procedure when an insufficient response after the first TACE occurred. All TACE procedures were performed successfully according to the Society of Interventional Radiology (SIR) guidelines 16.

### Follow-up schedule

The time interval between two consecutive TACE procedures was 1-3 months. Contrast-enhanced CT/MR was carried out 1 month before and 1-3 months after TACE. The treatment responses were assessed according to the modified response evaluation criteria in solid tumors (mRECIST).

### Definition of early TACE refractoriness

In accordance with the Japan Society of Hepatology (JSH) and the Liver Cancer Study Group of Japan (LCSGJ) consensus guidelines 6, 7, TACE refractoriness was recorded when any of the following criteria were met: (1) intrahepatic lesion: two or more consecutive ineffective responses was observed within the treated tumors (viable lesion >50%) or two or more consecutive progressions in the liver (including presence of new lesion compared to that before the previous TACE procedure), even after changing the chemotherapeutic agents or reanalysis of the feeding artery on response evaluation CT/MR after 1-3 months following adequately performed selective TACE; (2) alpha-fetoprotein (AFP): continuous elevated levels of tumor markers right after TACE; (3) vascular invasion was observed; and (4) extrahepatic spread was observed. According to the mRECIST criteria, the viable lesions >50% 17 is defined as the longest diameter of the viable tumor greater than 50% after TACE to that of the previous TACE. Presence of new lesions 17 is defined as the newly developed intrahepatic HCC lesions greater than 10 mm after TACE. Vascular invasion is defined as the newly developed vascular invasion after TACE 17, 18. The extrahepatic spread 17, 19 is defined as the newly developed extrahepatic metastasis after TACE.

In the present study, early TACE refractoriness was defined when patients met the TACE refractoriness criteria within the first two consecutive TACE sessions.

### Candidate predictors

The selection of candidate predictors was based on literature and other potential clinically meaningful parameters. The demographic characteristics included age and sex. The clinical data included the presence of an underlying liver disease, the Child-Pugh class, and the BCLC stage. The laboratory parameters included the initial AFP level (<400/≥400 ng/mL), neutrophil (NEUT) to lymphocyte (LY) ratio (NLR), platelet count (PLT), albumin (ALB, <35/≥35 g/L), and total bilirubin level (TBIL, <34.2/≥34.2 umol/L).

The radiological features included the tumor distribution (unilobar/bilobar), whether up-to-seven criteria were met, the number of tumors (solitary/2-3/>3), the size of the largest tumor (<50/50-100/>100 mm), vascularity of the largest tumor (hyper-/hypo- vascularity), and tumor enhancement pattern (homogeneous/heterogenous). Hypervascularity of the tumor was defined as an increase in the density/signal of the tumor compared to that of the surrounding liver tissue in the arterial phase in the CT/MR images 20. A heterogeneous enhancement pattern of the tumor was defined as a nonenhanced area within the tumor in the arterial phase, whereas a homogeneous enhancement pattern was defined as the lack of a nonenhanced area 21. Two abdominal radiologists with 22 and 19 years of experience in liver imaging who were blinded to all the clinical data independently reviewed the baseline CT/MR imaging data. The above-mentioned radiological features were assessed. The radiological results were finalized by discussion between the two radiologists.

### Process of model establishment

The process of RF model establishment was as follows:

#### Preprocessing

Due to the small sample size and the inherent sparsity of some features in the present study, the Min-Max Normalization method of feature scaling using Python software was used to preprocess dataset. This method will not change the essence of the data, and it also can speed up the calculation of RF model 22. Using randomized sampling method for splitting data, 158 of the total 227 patients were assigned to the training cohort for creating RF model and the remaining 69 patients were assigned to the validation cohort (approximately 30%).

#### Variable's selection (training cohort)

After preprocessing, the next step is to select final variables included in the model. Because a minimum events per variables (EPV) of 10 is required to train an adequate predictive model, the variables selection was based on the importance scores of RF algorithm to select maximal number of variables 23, 24. Unlike traditional variable selective method, such as univariate or multivariate chi-square test, RF algorithm can be more flexible, which calculate the change of Gini index of each variable 10. After selecting variables, the variation inflation factor (VIF) values (VIF<5) were calculated to measure the muliticolinearity among the selected variables.

#### Training and validation of the RF model

The RF model was developed using Python 3.6.5 with the “ensemble” module in the “sklearn” library, and this model was based on 5 potential predictors. The “GridSearchCV” module was used to adjust the parameters of the RF model automatically, and the best parameters were identified by this module, which included max depth = 5, min samples leaf = 10, min samples split = 5 and n-estimators = 80. In the process of adjusting the parameters, 5-fold cross-validation was used to prevent overfitting of the RF model and to maintain the stability and practicality of the model. The final two output nodes represented TACE non-refractoriness (=0) and TACE refractoriness (=1). The RF models were developed and validated with Python software (version 3.6.5, http://www.Python.org).

#### Performance measurement

Discrimination performance was assessed based on the receiver operating characteristic (ROC) curve and the corresponding AUC value. The calibration performance was validated by the Hosmer-Lemeshow test, in which a P value >0.05 indicated good performance.

### Statistical analysis

Statistical analysis was performed using a statistical software (SPSS version 20, International Business Machines Corporation, the United States) or Python software (version 3.6.5, http://www.Python.org). The continuous variables were expressed as means and standard deviation (SD) or as median and interquartile range (IQR). The differences in the continuous variables were compared using the independent sample t-test and rank-sum (Mann-Whitney) test. The categorical variables were shown as frequency and were compared using Pearson's chi-squared test or Fisher's exact test. A probability value of P<0.05 was deemed to indicate statistical significance.

To evaluate the inter-reader agreement of the radiological data between the two abdominal radiologists, either intraclass correlation coefficient (ICC) analysis (for numerical data) or the Kappa test (for categorical data) was performed. The agreement was classified as poor (ICC or Kappa value, 0-0.40), fair to good (ICC or Kappa value, 0.40-0.75), or excellent (ICC or Kappa value, >0.75).

## Results

### Patient characteristics

A total of 227 patients (204 males and 23 females, with a mean age of 56.4 ± 12.0 years) were included. Following a random split, 158 patients (70%) were assigned to a training cohort and the remaining 69 patients (30%) were assigned to a validation cohort. Among 227 patients, 131 patients (57.7%) in BCLC-0 to A and 96 patients (42.3%) in BCLC-B. The detailed demographic, radiological and laboratory characteristics are summarized in Table 1.

The inter-reader agreements of the radiological findings between the two radiologists were all excellent, with Kappa values of 0.949 (tumor distribution), 0.957 (number of tumors), 0.974 (vascularity of the largest tumor), and 0.931 (tumor enhancement pattern) and ICC values of 0.838 (diameter of the largest tumor).

### TACE refractoriness

The patterns of early TACE refractoriness in patients with HCC are illustrated in Table 2. Totally, there were 81 patients with early TACE refractoriness (81/227, 35.7%) in the entire study population. Among 81 patients, 53 patients were in the training set (53/158, 33.5%) and 28 patients in the validation set (28/69, 40.6%).

### Predictive variables

The detailed demographic, radiological and laboratory characteristics of the patients in the training and validation cohort are summarized in Table 3. In the training cohort, patients with or without early TACE refractoriness showed no difference in baseline characteristics except BCLC stage (P=0.043), AFP level (P=0.005), up-to-seven criteria (P<0.001), tumor distribution (P<0.001), number of tumors (P=0.001) the size of the largest tumor (P=0.035) and tumor enhancement pattern (P=0.005). In validation cohort, there were 4 variables with difference between patients with or without early TACE refractoriness, including BCLC stage (P<0.001), number of tumors (P<0.001), tumor distribution (P<0.001) and tumor enhancement pattern (P=0.022).

**Figure 2:**
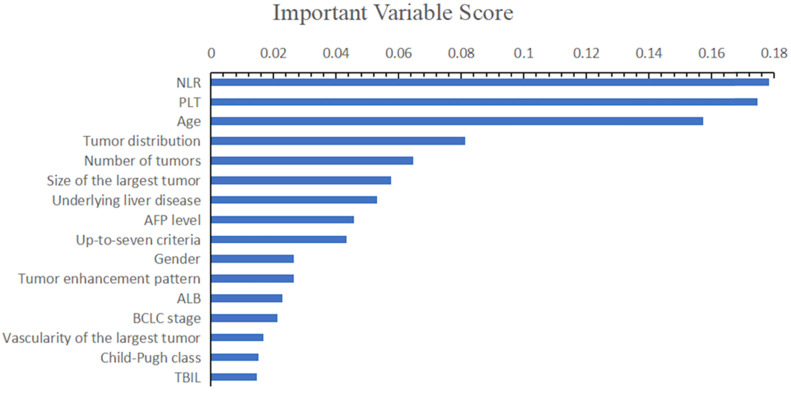
On the basis of important scores of the RF algorithm, NLR (score=0.178), PLT (score=0.175), patients' age (score=0.157), tumor distribution (score=0.081) and number of tumors (score=0.065) were selected as prognostic factors for predicting early TACE refractoriness in the training cohort.

**Figure 3:**
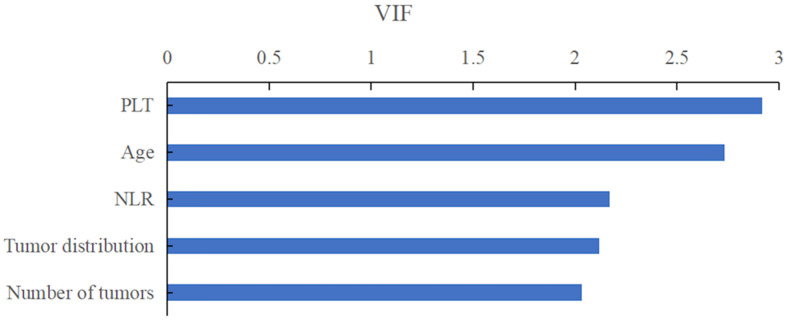
The VIF values of those variables were less than 5, therefore, the five variables showed no muliticolinearity and were included in the final predictive model.

On the basis of important scores generated by RF algorithm, NLR (score=0.178), PLT (score=0.175), patients' age (score=0.157), tumor distribution (score=0.081) and number of tumors (score=0.065) were selected as prognostic factors for predicting early TACE refractoriness in the training cohort (Figure 2). The VIF values of those variables were less than 5, therefore, the five variables showed no muliticolinearity and were included in the final predictive model (Figure 3).

### Performance of the predictive RF models

**Figure 4:**
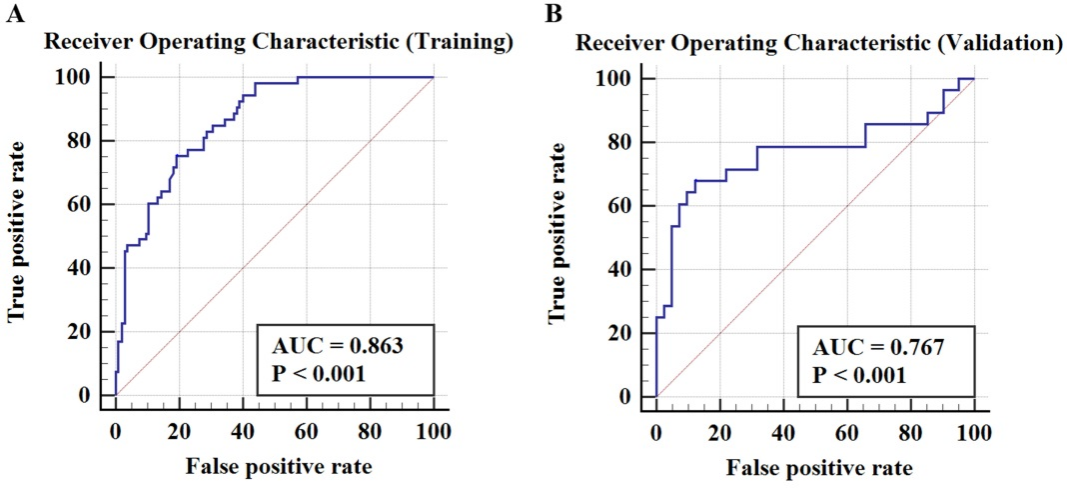
The ROC curve in training cohort and validation cohort. The AUCs in training cohort and validation cohort were 0.863 (95%CI, 0.800-0.913) and 0.767 (95%CI, 0.650-0.861), respectively.

Table 4 demonstrates a good discrimination of the proposed RF model for predicting early TACE refractoriness. The AUCs for the RF model in training cohort and validation cohort were 0.863 (95% CI: 0.800-0.913) and 0.767 (95% CI: 0.650-0.861), respectively. According to the confusion matrix, the RF model had a sensitivity of 75.5% and specificity of 81.0% in the training cohort, and a sensitivity of 67.9% and specificity of 87.8% in the validation cohort. The ROC curve of predicting early TACE refractoriness is shown in Figure 4. Moreover, satisfactory calibration was confirmed by the Hosmer-Lemeshow test, with P values of 0.538 and 0.068 in the training and validation cohort, respectively.

## Discussion

In the present study, the RF model has achieved a good performance in predicting early TACE refractoriness of HCC patients, with an AUCs of 0.863 and 0.767 in the training cohort and validation cohort, respectively. As suggested by the JSH and LCSGJ, patients with TACE refractoriness should switch to systemic therapy as soon as possible because repeat TACE is no longer effective, and systemic therapy may improve the patients' survival 6, 7. Therefore, a precise prediction of early TACE refractoriness is crucial 25. The results of the present study may be clinically significant because it provides a predictive model that differentiating patients who will occur early TACE refractoriness, permitting timely adjustment to the treatment planning.

In previous studies, several predictive models were established by traditional statistical methods such as the logistic regression (LR) model and Cox proportional hazards model 26, 27. The process of traditional model establishment is selecting the appropriate predictors, utilizing them for statistical analysis and ultimately deriving a multivariate predictive model. However, predictive models developed by traditional statistical methods are not reliable because the factors included in the models are too simple and utilize a low evidence level 28. With the development of ML algorithm, more and more ML algorithm-based predictive models have been created 29. Peng J et al. 30 have established a convolutional neural network model and Abajian A et al. 14 have created an RF model. To the best of our knowledge, this is the first study in the literature that using RF algorithm to predict early TACE refractoriness of HCC patients. The RF algorithm is a ML algorithm with multiple special decision trees (DTs) 10. Each DT typically comprises a root node, parent node and leaf node/terminal node. The training samples and input variables of each tree are randomly extracted from the training data sets with the bootstrap sampling method. Each tree gives a classified outcome, and the final result of the RF indicates the class based on the majority of votes from all the DTs. In order to achieve a good performance of RF model, the following procedures were performed in the present study. In the first step, the RF algorithm was used to select potential variables. Because the study population of the present study is relatively small, the number of variables included in the model should be limited to ensure the reliability. In the second step, RF algorithm was used to develop the model because RF algorithm has ability to explore and handle the potential nonlinear relationship between variable and results and prevent the overfitting of the model. In the last step, the combination of RF and five-fold cross validation method was used to prevent the mismatch between forecast value and the actual value, because the robust of the RF is estimated by cross validation.

In the present study, there were 5 predictors in the RF model, including patients' age, tumor number, tumor distribution, PLT, and NLR. Tumor number has been regarded as a predictive factor which is correlated with the treatment response after TACE, and the present study confirmed this finding. Because multiple tumors are usually associated with a more aggressive biological behavior of the tumor, therefore, it may have a higher probability incomplete embolization 31-33. Regarding tumor distribution, the present study showed that bilobar tumor distribution was a risk factor of early TACE refractoriness. Usually, bilobar tumor involvement is regarded as the intrahepatic metastasis of the primary lesion, it represents more aggressive nature of the tumor 34, which may result in an insufficient disease control. Additionally, the present study demonstrated that PLT and NLR were predictors of early refractoriness. Several inflammatory indices, such as PLT and NLR, have been investigated to predict the long-term prognosis of HCC patients 35, 36. However, regarding the early TACE refractoriness, their predictive potential has not been demonstrated yet, and the present study preliminary revealed that PLT and NLR were predictive factors of early TACE refractoriness.

The present study has several limitations. First, the sample size was relatively small which may lead to a statistical error. Therefore, the RF model could not be fully trained. A large sample size study should be conducted to improve the predictive performance. Second, this is a retrospective study, which may lead to selection bias. Third, the present study lacks an independent external validation cohort. Although the study population was from three independent hospital, an independent external study should be performed to confirm the reproducibility of RF model in the future.

In conclusion, the present RF model has a good predictive performance in patients with early TACE refractoriness. Once established, such a RF predictive model can easily be used in clinical practice and help determine the optimal patient care strategies.

## Figures and Tables

**Table 1:** The baseline demographic, radiological and laboratorial characteristics

Characteristics	Overall (n= 227)	Training cohort (n=158)	Validation cohort (n=69)	P
Age (years)	56.4±12.0	56.5±12.4	56.1±11.1	0.820
**Gender (%)**				0.056
male	204 (89.9)	138 (87.3)	66 (95.7)	
female	23 (10.1)	20 (12.7)	3 (4.3)	
**Underlying liver disease (%)**				0.483
None	29 (12.8)	20 (12.7)	9 (13.0)	
HBV	182 (80.2)	129 (81.6)	53 (76.8)	
Others	16 (7.0)	9 (5.7)	7 (10.1)	
**Child-Pugh class (%)**				0.012
A	197 (86.8)	143 (90.5)	54 (78.3)	
B	30 (13.2)	15 (9.5)	15 (21.7)	
**BCLC stage (%)**				0.265
0 -A	131 (57.7)	95 (60.1)	36 (52.2)	
B	96 (42.3)	63 (39.9)	33 (47.8)	
**AFP level (%)**				0.267
<400 ng/mL	139 (61.2)	93 (58.9)	46 (66.7)	
≥400 ng/mL	88 (38.8)	65 (41.1)	23 (33.3)	
NEUT (×10^9^/L, IQR)	3.01 (2.44)	3.00 (2.72)	3.01 (2.01)	0.801
LY (×10^9^/L, IQR)	1.04(0.84)	1.02 (0.85)	1.09 (0.76)	0.415
NLR (IQR)	2.93 (3.55)	3.01 (3.66)	2.70 (3.14)	0.267
PLT (×10^9^/L, IQR)	141 (113)	145 (113)	128 (109)	0.150
**ALB (%)**				0.485
<35 g/L	62 (27.3)	41 (25.9)	21 (30.4)	
≥35 g/L	165 (72.7)	117 (74.1)	48 (69.6)	
**TBIL (%)**				0.562
<34.2 umol/L	212 (93.4)	146 (92.4)	66 (95.7)	
≥34.2 umol/L	15 (6.6)	12 (7.6)	3 (4.3)	
**Up-to-seven criteria (%)**				0.199
within	90 (39.6)	67 (42.4)	23 (33.3)	
beyond	137 (60.4)	91 (57.6)	46 (66.7)	
**Tumor distribution (%)**				0.533
unilobar	145 (63.9)	103 (65.2)	42 (60.9)	
bilobar	82 (36.1)	55 (34.8)	27 (39.1)	
**Number of tumors (%)**				0.120
solitary	128 (56.4)	95 (60.1)	33 (47.8)	
2-3	59 (26.0)	35 (22.2)	24 (34.8)	
>3	40 (17.6)	28 (17.7)	12 (17.4)	
**Size of the largest tumor (%)**				0.759
<50 mm	88 (38.8)	63 (39.9)	25 (36.2)	
50-100 mm	84 (37.0)	56 (35.4)	28 (40.6)	
>100 mm	55 (24.2)	39 (24.7)	16 (23.2)	
**Vascularity of the largest tumor (%)**				0.636
hyper-vascularity	204 (89.9)	141 (89.2)	63 (91.3)	
hypo-vascularity	23 (10.1)	17 (10.8)	6 (8.7)	
**Tumor enhancement pattern (%)**				0.423
homogeneous	30 (13.2)	19 (12.0)	11 (15.9)	
heterogenous	197 (86.8)	139 (88.0)	58 (84.1)	
**TACE refractoriness (%)**				0.309
presence	81 (35.7)	53 (33.5)	28 (40.6)	
absence	146 (64.3)	105 (66.5)	41 (59.4)	

Abbreviations: HBV, hepatitis B virus; BCLC, Barcelona-Clinic-Liver-Cancer; AFP, alpha-fetoprotein; NEUT, neutrophil count; IQR, inter-quartile range; LY, lymphocyte count; NLR, NEUT to LY ratio; PLT, platelet count; ALB, albumin; TBIL, total bilirubin; TACE, transarterial chemoembolization.

**Table 2:** The patterns of early TACE refractoriness in patients with HCC

Characteristics	Total (n=81)	Training set (n=53)	Validation set (n=28)	P value
Viable lesions > 50%, n (%)	51 (63.0)	32 (60.4)	19 (67.9)	0.507
Presence of new lesions, n (%)	11 (13.6)	7 (13.2)	4 (14.3)	1.000
Elevation of AFP, n (%)	35 (43.2)	22 (41.5)	13 (46.4)	0.671
Vascular invasion, n (%)	9 (11.1)	7 (13.2)	2 (7.1)	0.487
Extrahepatic spread, n (%)	5 (6.2)	5 (9.4)	0	-

Abbreviations: TACE, transarterial chemoembolization; HCC, hepatocellular carcinoma; AFP, alpha-fetoprotein.

**Table 3:** The baseline characteristics of patients in training set (n=158) and validation set (n=69)

Characteristics	Training set (n=158)			Validation set (n=69)		
	TACE refractoriness (n=53)	TACE non-refractoriness (n=105)	P	TACE refractoriness (n=28)	TACE non-refractoriness (n=41)	P
Age (years)	55.9±12.4	56.8±12.4	0.684	55.9±12.6	56.3±10.1	0.874
**Gender (%)**			0.883			0.562
male	46 (86.8)	92 (87.6)		26 (92.9)	40 (97.6)	
female	7 (13.2)	13 (12.4)		2 (7.1)	1 (2.4)	
**Underlying liver disease (%)**			0.160			0.438
None	10 (18.9)	10 (9.5)		2 (7.1)	7 (17.1)	
HBV	39 (73.6)	90 (85.7)		24 (85.7)	29 (70.7)	
Others	4 (7.5)	5 (4.8)		2 (7.1)	5 (12.2)	
**Child-Pugh class (%)**			0.243			0.256
A	50 (94.3)	93 (88.6)		20 (71.4)	34 (82.9)	
B	3 (5.7)	12 (11.4)		8 (28.6)	7 (17.1)	
**BCLC stage (%)**			0.043			<0.001
0-A	26 (49.1)	69 (65.7)		6 (21.4)	30 (73.2)	
B	27 (50.9)	36 (34.3)		22 (78.6)	11 (26.8)	
**AFP level (%)**			0.005			0.729
<400 ng/mL	23 (43.4)	70 (66.7)		18 (64.3)	28 (68.3)	
≥400 ng/mL	30 (56.6)	35 (33.3)		10 (35.7)	13 (31.7)	
NLR (IQR)	3.26 (4.28)	3.00 (3.46)	0.518	3.00 (3.17)	2.41 (2.73)	0.095
PLT (×10^9^/L, IQR)	167 (99)	138 (114)	0.053	132 (101)	128 (107)	0.793
**ALB (%)**			0.772			0.799
<35 g/L	13 (24.5)	28 (26.7)		9 (32.1)	12 (29.3)	
≥35 g/L	40 (75.5)	77 (73.3)		19 (67.9)	29 (70.7)	
**TBIL (%)**			0.219			1.000
<34.2 umol/L	47 (88.7)	99 (94.3)		27 (96.4)	39 (95.1)	
≥34.2 umol/L	6 (11.3)	6 (5.7)		1 (3.6)	2 (4.9)	
**Up-to-seven criteria (%)**			<0.001			0.083
within	12 (22.6)	55 (52.4)		6 (21.4)	17 (41.5)	
beyond	41 (77.4)	50 (47.6)		22 (78.6)	24 (58.5)	
**Tumor distribution (%)**			<0.001			<0.001
unilobar	22 (41.5)	81 (77.1)		9 (32.1)	33 (80.5)	
bilobar	31 (58.5)	24 (22.9)		19 (67.9)	8 (19.5)	
**Number of tumors (%)**			0.001			<0.001
solitary	26 (49.1)	69 (65.7)		6 (21.4)	27 (65.9)	
2-3	9 (17.0)	26 (24.8)		13 (46.4)	11 (26.8)	
>3	18 (34.0)	10 (9.5)		9 (32.1)	3 (7.3)	
**Size of the largest tumor (%)**			0.035			0.395
<50 mm	15 (28.3)	48 (45.7)		8 (28.6)	17 (41.5)	
50-100 mm	19 (35.8)	37 (35.2)		14 (50.0)	14 (34.1)	
>100 mm	19 (35.8)	20 (19.0)		6 (21.4)	10 (24.4)	
**Vascularity of the largest tumor (%)**			0.702			-
hyper-vascularity	48 (90.6)	93 (88.6)		28 (100)	35 (85.4)	
hypo-vascularity	5 (9.4)	12 (11.4)		0	6 (14.6)	
**Tumor enhancement pattern (%)**			0.005			0.022
homogeneous	1 (1.9)	18 (17.1)		1 (3.6)	10 (24.4)	
heterogenous	52 (98.1)	87 (82.9)		27 (96.4)	31 (75.6)	

Abbreviations: TACE, transarterial chemoembolization; HBV, hepatitis B virus; BCLC, Barcelona Clinic Liver Cancer; AFP, alpha-fetoprotein; IQR, inter-quartile range; NLR, neutrophil to lymphocyte ratio; PLT, platelet count; ALB, albumin; TBIL, total bilirub.

**Table 4:** Performance of the RF model in predicting early TACE refractoriness

	Training cohort	Validation cohort
AUC (95% CI)	0.863 (0.800, 0.913)	0.767 (0.650-0.861)
PPV (%)	66.6	79.2
NPV (%)	86.8	80.0
Sensitivity (%)	75.5	67.9
Specificity (%)	81.0	87.8

Abbreviations: RF, random forest model; TACE, transarterial chemoembolization; AUC, area under curve; CI, confidence interval; PPV, positive predictive value; NPV, negative predictive value.
